# Increased Brain-Specific MiR-9 and MiR-124 in the Serum Exosomes of Acute Ischemic Stroke Patients

**DOI:** 10.1371/journal.pone.0163645

**Published:** 2016-09-23

**Authors:** Qiuhong Ji, Yuhua Ji, Jingwen Peng, Xin Zhou, Xinya Chen, Heng Zhao, Tian Xu, Ling Chen, Yun Xu

**Affiliations:** 1 Department of Neurology, Drum Tower Hospital of Nanjing Medical University, Nanjing, Jiangsu, China; 2 Department of Neurology, Affiliated Hospital of Nantong University, Nantong, China; 3 Institute of Immunology, College of Life Science and Technology, Jinan University, Guangdong, China; 4 Key Laboratory of Neuroregeneration, Nantong University, Nantong, China; 5 Department of Neurosurgery, Stanford University School of Medicine, Stanford, California, United States of America; 6 Department of Physiology, Nanjing Medical University, Nanjing, China; Universita degli Studi di Torino, ITALY

## Abstract

The aims of this study were to examine the alternation in serum exosome concentrations and the levels of serum exosomal miR-9 and miR-124, two brain-specific miRNAs, in acute ischemic stroke (AIS) patients and to explore the predictive values of these miRNAs for AIS diagnosis and damage evaluation. Sixty-five patients with AIS at the acute stage were enrolled and 66 non-stroke volunteers served as controls. Serum exosomes isolated by ExoQuick precipitations were characterized by transmission electron microscopy, nanoparticle-tracking analysis and western blotting. The levels of exosomal miR-9 and miR-124 were determined by real-time quantitative PCR. Compared with controls, the concentration of serum exosomes and the median levels of serum exosomal miR-9 and miR-124 were significantly higher in AIS patients (p<0.01). The levels of both miR-9 and miR-124 were positively correlated with National Institutes of Health Stroke Scale (NIHSS) scores, infarct volumes and serum concentrations of IL-6. The areas under the curve for exosomal miR-9 and miR-124 were 0.8026 and 0.6976, respectively. This proof of concept study suggests that serum exosomal miR-9 and miR-124 are promising biomarkers for diagnosing AIS and evaluating the degree of damage caused by ischemic injury. However, further studies are needed to explore the potential roles of the exosomes released from brain tissues in post stroke complications.

## Introduction

Stroke is a leading cause of death and long-term adult disability worldwide, and ischemic strokes account for more than 80% of stroke cases. Ischemic stokes occur when a vessel supplying blood to the brain is blocked. Early and accurate diagnosis of ischemic strokes is of critical importance for clinical therapy and would greatly improve the outcome and prognosis [1]. In the past decade, notable progress has been made toward understanding the physiopathology and biochemistry of stroke, and a number of ischemic injury-related proteins have been identified, such as calcium binding protein B (S100B), neuron-specific enolase (NSE), myelin basic protein (MBP) and glial fibrillary acidic protein (GFAP) [1]. However, a sensitive biomarker, such as troponin in the assessment of myocardial infarction [2], is still lacking, and the methods currently available for diagnosis and prognosis of cerebral ischemia need to be improved.

MicroRNAs (miRNAs), which are endogenous small (~22nt) single-stranded non-coding RNAs, play important roles in regulating various biological processes by complementarily binding to 3`untranslated regions (UTRs) of targeted mRNAs [3]. They are expressed in a tissue-specific manner, such that a single miRNA or several miRNAs can dominate all the miRNAs of a tissue in terms of abundance [4]. In addition to intercellular distribution, miRNAs are ubiquitous in all types of body fluid, including serum, plasma and saliva [5]. These circulating miRNAs are packaged in exosomes or form complexes with microparticles or lipoproteins and are therefore remarkably stable [6]. Owing to their tissue specificity, relative stability and abundance in bodily fluids, miRNAs are considered to be sensitive plasma biomarkers for tissue injury [7].

Exosomes are 30–100nm membrane-derived vesicles released by most cell types and exist in almost all body fluids. Exosomes contain a variety of proteins, nucleic acids and lipids derived from their host [8]. These small vesicles carrying cargos from their host are considered to be novel and useful sources for gaining pathogenic information and identifying biomarkers, especially for tissues that are not accessible to direct examination, such as the central nervous system [9]. More importantly, unlike the circulating miRNAs packaged in protein complexes, exosomes can mediate cell-to-cell communication by delivering miRNAs cargos [8].

MiR-9 and miR-124 are two brain-specific miRNAs that are highly expressed in the developing and adult vertebrate brain [4, 10]. They are wholly homologous in mice, rats and humans and are involved in a broad spectrum of biological functions in the central nervous system, such as neurodegeneration, neuroimmune disorders and stroke [4, 10]. In the present study, we examined the effects of acute ischemic stroke on the exosomal expression of these two miRNAs and discussed the potential roles of exosomes derived from the brain in the pathophysiology of stoke.

## Materials and Methods

### Standard protocol approvals and patient consents

The ethics committee of the Affiliated Hospital of Nantong University approved this research protocol, and participants or their legally authorized representatives provided written informed consent.

### Subject demographics and clinical characteristics

AIS patients were recruited among those admitted to the Department of Neurology of the Affiliated Hospital of Nantong University between September 2014 and August 2015. Either magnetic resonance imaging (MRI) or computed tomography (CT) imaging of the brain confirmed ischemic stroke. Experienced neurologists determined the neurological deficits by using the NIHSS within 24 hours after the stroke onset. Patients with intracerebral hemorrhage or unknown etiology were excluded. The mean time of enrollment blood draw was 16.5 hours. Non-stroke controls were recruited from those who underwent an annual medical examination at our hospital. The demographics and clinical characteristics of the 65 AIS patients and 66 non-stroke controls are provided in Table 1.

**Table 1:** Baseline characteristics of study participants.

Parameter	AIS patients (n = 65)	Non-stroke controls (n = 66)	P
Age	64 (54–70)	60(53–64)	0.13
Male	40(61.5%)	36(54.5%)	0.42
Stroke risk factors			
Hypertension	43(66.2%)	35(53%)	0.13
Diabetes mellitus	18(27.7%)	12(18.2%)	0.20
Atrial Fibrillation	14(21.5%)	8(12.1%)	0.15
Hyperlipidemia	18(27.7%)	16(24.2%)	0.66
Coronary heart disease	4(6.2%)	4(6.1%)	0.98
Baseline median NIHSS score	8	0	<0.01
25th–75th percentile	6–11	-	-
Baseline DWI median volume (cm^3^)	18.58	0	<0.01
25th–75th percentile (cm^3^)	9.79–30.48	-	-

All continuous data are expressed as medians (range). Categorical variables are expressed as values (percentages)

### MRI and lesion volume assessment

MRI was performed with a 3.0-T clinical whole-body MR scanner. The edge of the hyperintense signal on each slice was manually outlined, and the volume was determined by multiplying the area of diffusion hyperintensity by the slice thickness plus the interslice gap. The total lesion volume was calculated by summing the lesion volume on each slice. Two independent observers measured the DWI lesion volumes, and the results were averaged for further analysis.

### Serum Collection

Five milliliters of venous blood were collected into plain tubes, allowed to clot at 37°C for 30 min and refrigerated at 4°C for 2 h, and then were centrifuged at 1,600 g for 10 min. Hemolysis samples were excluded at this step. Clear serum was transferred to fresh tubes and centrifuged at 20,000 g for another 10 min at 4°C. This step was used to remove cell debris, apoptotic bodies and blood platelets that were packed with large amounts of miRNAs [11, 12]. Then, the supernatant was transferred to labeled EP tubes and stored at -80° until analysis.

### Exosomes isolation

To isolate exosomes, -80°C stored serum was thawed on ice and centrifuged at 21,000 g for 15 min at 4°C, and the supernatant was transferred to a new tube. According to the recommended protocol, a one-fourth volume of ExoQuick Solution (System Biosciences Inc., Mountain View, CA, USA) was added and incubated at 4°C for 2 hours after brief up-and -down mix. Then, the mixture was centrifuged at 1,500 g for 30 minutes and the supernatant was removed by careful aspiration. Centrifugation for another 5 min was performed to remove the residual liquid. The exosome-containing pellet was subsequently re-suspended in nuclease-free water.

### Nanoparticle-tracking analysis (NTA)

We used a NanoSight NS300 Instrument with a 405-nm laser (NanoSight Ltd, Amesbury, United Kingdom) to analyze the size distribution and quantify the exosome preparations. Isolated exosomes were diluted 1,000 times in particle-free PBS to obtain approximately 5 × 10^8^ exosomes/ml, and they were then injected into the NanoSight sample chamber. The size distributions and vesicle concentrations were assessed with Nanoparticle Tracking Analysis (NTA) software.

### Transmission electron microscopy (TEM)

For TEM morphology investigation, samples were prepared as described by Thery et al. [13]. Briefly, the exosomal fraction was mixed with an equal volume of 4% paraformaldehyde, and 10 μl of the samples were then transferred onto Formvar/carbon-coated nickel grids, and the membranes were allowed to adsorb for 20 min at room temperature. After a quick wash with 100 μl drops of PBS, the grids were fixed with 50 μl 1% w/v glutaraldehyde for 5 min and then washed with distilled water for seven times. The samples were contrasted with 4% w/v uranyl acetate (UA) for 5min and embedded in a mixture of 4% UA and 2% methylcellulose (100:900) solution for 10 min on ice. The excess fluid was blotted and air-dried, and the grids were examined using TEM (JEM-2100 JEOL, Tokyo, Japan).

### Western blotting analysis

Exosomal protein was extracted with RIPA lysis buffer (Pierce, Rockford, IL, USA). The total protein concentration was quantified with a BCA protein assay kit (Pierce, Rockford, IL, USA). Approximately 40 μg of total protein was separated by 12% sodium dodecyl sulfate-polyacrylamide gel electrophoresis and transferred to a PVDF membrane. The membrane was blocked for 1 h at room temperature with 5% bovine serum albumin, then immunoblotted with anti-CD9, anti-CD81 (Abcam, Cambridge, UK), and anti-CD63 antibodies (System Biosciences Inc., Mountain View, CA, USA). The proteins were detected by using enhanced chemiluminescence (Pierce, Rockford, IL, USA).

### RNA extraction, quantification and quantitative real-time PCR (qPCR)

Total RNA was extracted from the serum exosomes using an Exosome RNA Purification Kit (System Biosciences Inc., Mountain View, CA, USA) according to the manufacturer’s protocol. Twenty-five fmol synthetic cel-miR-39 (RiboBio, Guangzhou, China) was added as a spike-in control. The concentration and integrity of isolated RNA were evaluated with a Bioanalyzer 2100 instrument (Agilent Technologies, Santa Clara, CA, USA). miDETECT A Track™ miRNA qRT-PCR kits, containing miRNA-specific forward primer and the sequence complementary to the poly(T) adapter as the reverse primer, were purchased from Riobio (Riobio, Guangzhou, China). Total exosome RNAs extracted from 250 μl serum were polyadenylated and reverse-transcribed with a poly (T) adapter into cDNAs. The expression of miR-124 and miR-9 was detected by using Perfect Real Time kit (SYBR® Premix ExTaq™ II,TaKaRa, Dalian, China) and a CFX96 PCR system (Bio-Rad, Hercules, CA) according to the manufacturer’s instructions and all reactions were run in triplicate.

The raw qPCR data of miRNAs were normalized to spiked cel-miR-39 as described previously [11]. Target _normalized_ = Target _raw_—(Control _raw_—Control _median run_), where “Target” is the miRNA to be normalized; “Control” is cel-miR-39, the mean Cq of cel-miR-39; “raw” is the instrument Cq result for that sample, and “median run” is the median Cq of all 131 raw results for the mean Cq of cel-miR-39.

### Determination of serum IL-6 concentrations

The serum concentration of IL-6 was measured with a high-sensitivity ELISA kit (Blue gene, Shanghai, China). The recommended serum concentration of IL-6 in non-stroke individuals is less than 6.4 pg/ml. Values below the detection limit (0.08 pg/ml) were assigned a value equal to half the detection limit.

### Statistical analysis

Statistical analysis was performed by using GraphPad Prism 5 (GraphPad Software Inc., La Jolla, CA, USA). Categorical variables expressed as numbers and percentages were obtained for demographic data, and a chi-square test was used to compare categorical variables between groups. Continuous variables are presented as medians (with the interquartile range), and a Wilcoxon test was used to compare continuous variables. Correlations between the variables were assessed by using the Spearman's correlation coefficient. Receiver-operating-characteristic (ROC) curves were constructed to assess the sensitivity and specificity of the miR-9 and miR-124 measurements and to compare their ability to diagnose acute ischemic stroke. All hypothesis testing was two-tailed, and p-values of less than 0.01 were considered to be statistically significant.

## Results

### Characterization of serum exosomes isolated from AIS patients

Obtaining a sufficient amounts of "real" exosomes is a prerequisite for an exosome study. Ultracentrifugation remains the golden standard for exosomes isolation; however, owing to the limited volume of serum and the lower yield after repeated ultracentrifugation, we chose ExoQuick, which is a method based on precipitation, to isolate serum exosomes [14]. According to the minimal experimental requirements for the definition of extracellular vesicles, we characterized the preparation on the basis of morphology, size and membrane composition [15].

**Fig 1:**
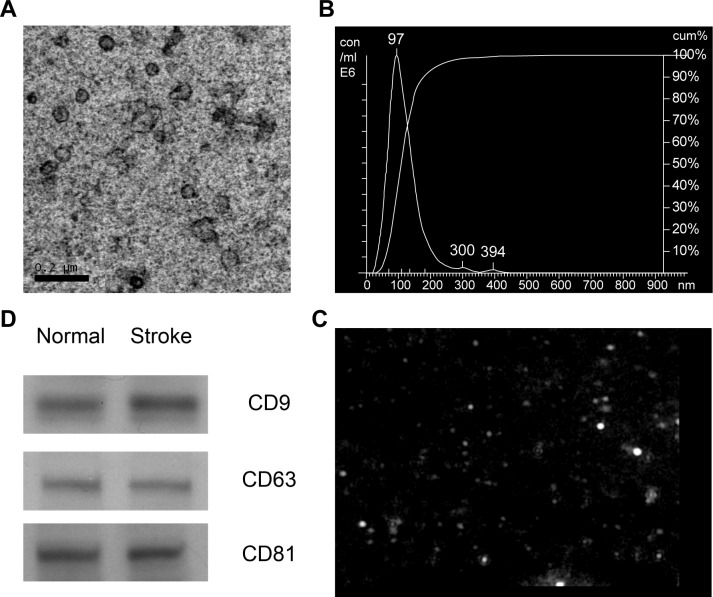
Characterization of isolated exosomes from human serum. (A) The TEM image shows the spherical morphology of exosomes with a diameter of approximately 100 nm, bar = 200 nm. Representative images show the size distribution (B) and morphology (C) of serum exosomes measured by NTA. (D) Western blotting analysis of exosome markers, CD9, CD63 and CD81, in the serum exosomes from non-stroke controls and stroke patients.

Consistently with the characteristics of exosomes [16], precipitated serum microvesicles displayed spherical structures with a diameter of approximately 100 nm (Fig 1A). NanoSight analysis showed that the mode size of isolated serum exosomes was 98 nm, ranging from 30 nm to 150 nm (Fig 1C and 1D). The expression of three commonly used exosomal markers, CD81, CD9 and CD63, was detected by western blotting. Antibodies against CD81 probed 26 kDa bands, the expected molecular weight of CD81, and similar results were obtained for CD9 and CD63 (Fig 1B). In summary, the obtained microvesicles exhibited the major characteristics of exosomes, and this isolation method was used in all of the subsequent experiments.

### The concentration of serum exosomes and the levels of serum exosomal miR-9 and miR-124 were significantly elevated in stroke patients

**Fig 2:**
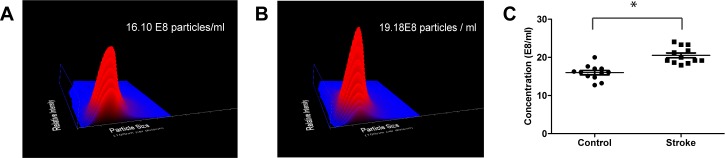
Comparison of serum exosomal concentrations of non-stroke controls and the stroke group. Representative plots of NTA analysis of serum exosomes from a non-stroke individual (A) and an AIS patient (B). The indicated concentration is the value for 1,000 diluted samples. (C). Statistical analysis of the serum exosomal concentrations of AIS patients and non-stroke controls, *, p<0.01.

Serum microvesicles (MVs) concentrations are modulated by multiple factors, such as inflammation and injury [9]; however, whether AIS injury affects the serum exosome concentrations remains unknown. The serum exosome concentration of 11 randomly selected AIS patients and corresponding non-stroke controls were examined by NTA. As shown in Fig 2A–2C, the serum exosome concentrations of AIS patients were significantly higher than those of non-stroke individuals, and the mean serum exosome concentrations increased from 16.00E8 to 20.54E8 (p<0.01).

Next, the relative abundance of serum exosomal miR-9 and miR-124 was detected by using real time qPCR. Because there is a lack of housekeeping genes that are suitable for use as normalization control in studies of circulating exosomal microRNAs, we established a practical workflow to ensure that the PCR data obtained from different experiments and individuals were comparable. First, total RNA was extracted from exosomes precipitated from the same volume serum (250 μl) and was eluted in the same volume of RNase free water. The same volume of RNA solution was then used for 3`-extension with a poly (A) tail and subsequent reverse transcription and qPCR. Second, 25 fmol of Cel-miR-39 was spiked in as an external control and for the following data normalization. Finally, in our initial experiment we determined the relative stability of miR-451, an abundant microRNA present in red blood cells, which was monitored as an internal control.

**Fig 3:**
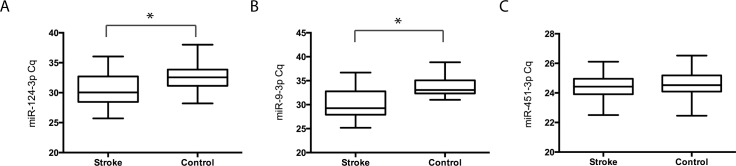
Comparison of levels of serum exosomal miR-9, miR-124 and miR-451 between stroke patients and non-stroke controls. Relative serum exosomal miR-9 (A), miR-124 (B) and miR-451 (C) levels between the stroke group (n = 65) and non-stroke control groups (n = 66). The y-axis depicts Cq values normalized to cel-miR-39. The black horizontal line in the box represents the median value of the group. *, p<0.01.

As illustrated in Fig 3A and 3B, compared with the control group, the levels of both miR-9 and miR-124 were significantly increased in the stroke group (p<0.01). The change in miR-9 was relatively higher than that in miR-124, because the median Cq values of miR-9 and miR-124 in the stroke group were approximately 4 and 2 cycles lower than those in the control group, that is a 16-fold and 4-fold increased in AIS patients, respectively. As expected, no significant differences were observed in the exosomal level of miR-451 (p>0.1) (Fig 3C).

### Correlations between the levels of serum exosomal miR-9, miR-124 and NIHSS scores and infarct volume

**Fig 4:**
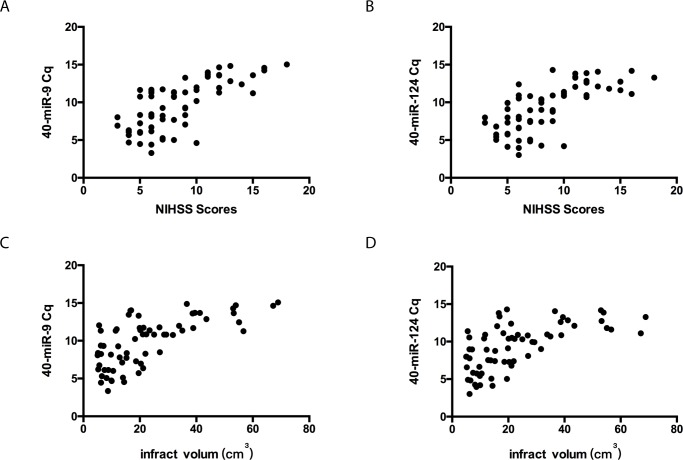
**Correlations between the levels of serum exosomal miR-9 (A), miR-124 (B) and the NIHSS score in 65 AIS patients at an acute stage. Correlations between the levels of serum exosomal miR-9 (C), miR-124 (D) and the infarction volume in 65 stroke patients at an acute stage.** The scatter plots show significant positive correlations between the serum exosomal miR-9 and miR-124 levels (expressed as 40-Delta Cq) and NIHSS scores and infarct volumes.

To determine the diagnostic value of miR-9 and miR-124 in the acute stage of ischemic stroke, the correlations between the levels of these two microRNAs and NIHSS scores and infarct volume were examined. Consistently with results from previous report [17], NIHSS scores were positively correlated with infarct volume in the AIS patients (r = 0.7856, p<0.01) (S1 Fig). More importantly, significant correlations were found between the levels of miR-9 and miR-124 and NIHSS scores (r = 0.7126 and 0.6825 respectively, p < 0.01) (Fig 4A and 4B). Similarly, correlations were observed between the expression levels of miR-9 and miR-124 and the infarct volume (r = 0.6768 and 0.6312, respectively, p<0.01) (Fig 4C and 4D).

### Correlations between the levels of serum exosomal miR-9, miR-124 and serum IL-6

**Fig 5:**
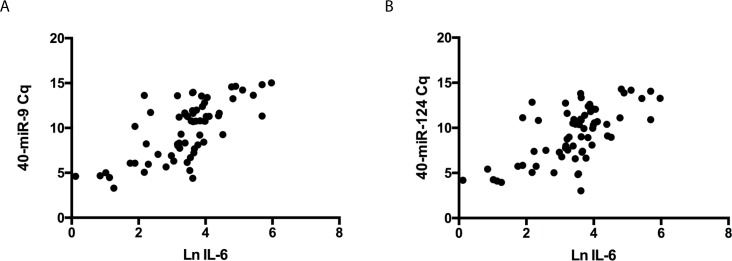
**Correlations between the levels of serum exosomal miR-9 (A) and miR-124 (B) and the serum concentrations of IL-6 in stroke patients.** The scatter plots show significant positive correlations between the serum exosomal miR-9 and miR-124 levels (expressed as 40-Delta Cq) and Ln transferred IL-6 concentrations.

IL-6 is a pro-inflammatory cytokine that is released early after the onset of brain injury. The level of serum IL-6 can reflect the degree of brain ischemic damage and correlates with stroke lesion volume [18–20]. To further evaluate the relationship between the exosomal miRNAs and brain ischemic damage, correlations between the levels of miR-9 and miR-124 and the concentration of serum IL-6 were analyzed. As shown in Fig 5, the levels of both miR-9 and miR-124 showed positive correlations with serum IL-6 (r = 0.6980 and 0.6550, respectively) (p<0.01).

### ROC analyses of the diagnostic value of exosomal miR-9 and miR-124 for AIS

**Fig 6:**
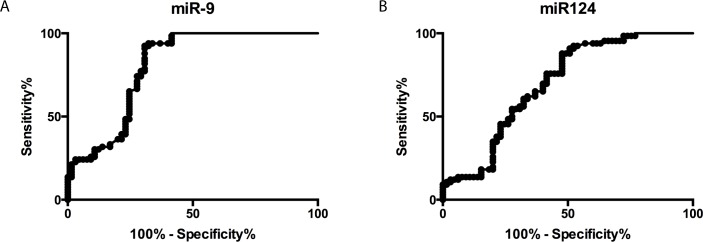
Diagnostic value of serum exosomal miR-9 and miR-124 levels in AIS. ROC curves based on exosomal miR-9 (A) and miR-124 (B) levels to differentiate AIS patients (n = 65) from non-stroke individuals (n = 66). The areas under the curve (AUC) are 0.8026 (95% CI: 0.7235–0.8816) and 0.6976 (95% CI: 0.6506–0.7895).

To evaluate the diagnostic value of exosomal miR-9 and miR-124 for AIS, ROC curves were generated to discriminate AIS patients from non-stroke controls. The areas under the curve (AUC) for exosomal miR-9 and miR-124 were 0.8026 (95% CI: 0.7235–0.8816) and 0.6976 (95% CI: 0.6506–0.7895), respectively. These findings suggested that both miR-9 and miR-124 may be sensitive biomarkers that can identify the AIS patients from non-stroke individuals (Fig 6) and miR-9 showed a better diagnostic value than miR-124.

## Discussion

In the present study, we successfully isolated and characterized serum exosomes from AIS patients and explored the effects of acute ischemic brain injury on serum exosome concentrations and levels of serum exosomal miR-9 and miR-124, two brain-specific microRNAs. Compared with non-stroke controls, in patients with acute ischemic injury, we found that both the concentrations of serum exosomes and the levels of serum exosomal miR-9 and miR-124 were significantly elevated. Furthermore, this increase was coordinated with NIHSS scores, infarct volume and the overexpression of pro-inflammatory cytokine IL-6, thus suggesting a close relationship between the levels of serum exosomal miR-9 and miR-124 and the degree of brain damage. To our knowledge, this is the first study exploring the serum exosomal miRNAs in AIS patients.

The number of MVs released by many cell types often increases after cellular activation, and is considered to be a novel biomarkers for neurological disorders [9]. Using flow cytometry and surface marker staining for platelets and endothelial cells, Kuriyama and Jung have found increased plasma MVs during cerebral ischemia and a positive connection between the number of MVs derived from endothelial cells and platelets, the occurrence of ischemic stroke and the extent of cerebral ischemia [21, 22]. Because FACS is limited in its ability to perform microparticle measurements (diameter less than 0.1 micrometer) [23], the MVs detected in previous studies are mainly ectosomes (shed vesicle) rather than exosomes. In the present study, we demonstrated an increased concentration of serum exosomes in AIS patients by using NTA (Fig 2). On the basis of the significantly elevated levels of brain-specific miR-9 and miR-124 in serum exosomes, we proposed that part of these increased exosomes in the peripheral blood of AIS patients may derive from brain tissues. It is also reasonable to postulate that there may be more exosomes derived from platelet and endothelial cells in the sera of AIS patients. Novel techniques are needed to efficiently and conveniently identify exosomes derived from specific cells or brain tissues, whereby the relationship between these cellular or tissue specific exosomes and neurological disorders can be directly determined.

Owing to their high stability, relative tissue specificity, association with disease states, presence in readily detectable concentrations, ease of manipulation and measurement sensitivity, circulating miRNAs are considered to be promising sources for the discovery of sensitive and specific biomarkers of a broad spectrum of diseases [24]. A number of studies have already been carried out to screen miRNAs from peripheral blood and plasma for stroke diagnosis and prognosis and have revealed aberrant expression of miR-145, miR-21, miR-210, miR-107, let-7b and let-7e in the peripheral blood of ischemic stroke patients [25, 26]. However, most of the identified miRNAs lack brain specificity; for instance, miR-145 is a modulator of a smooth muscle phenotype and is predominantly expressed in the heart [27, 28], and miR-21 has a nonspecific distribution [4]. This lack of specificity is similar to observations in cancer biomarker studies. Many identified circulating miRNA biomarkers for cancers are highly expressed in blood cells, and Pritchard et al have suggested that these identified biomarkers reflected a blood cell-based phenomenon rather than a cancer-specific origin [29]. Therefore, these miRNAs, which were identified to be associated with stroke, may reflect the multiple effects of brain damage on the peripheral system and organs, such as heart and endothelial cells.

MiR-124 is the most abundant miRNA in the brain and is expressed in almost all brain regions [7]. MiR-9 is one of the most highly expressed miRNAs in the developing and adult vertebrate brain and is a versatile regulator of neurogenesis [30]. Existing clinical and animal studies have reported higher plasma concentrations of miR-124 after ischemic brain injury [7, 10] [31], but there are few studies reporting miR-9 levels in stroke. Recent studies have shown that the expression of circulating miR-9 is coordinated with the severity of acute spinal cord injury [32] and the evolution of neural cell death [33], and it is considered to be a novel indicator of neural damage and neurotoxicity. In line with these reports and the observation that the majority of the miRNAs in serum are concentrated in exosomes [34], we found the significantly elevated levels of both serum exosomal miR-124 and miR-9 in AIS patients, and their levels were positively correlated with NIHSS scores and infarct volume (Fig 4). Moreover, miR-9 may be a novel noninvasive biomarker for AIS with higher sensitive/specificity than miR-124. Inflammation plays an important role in the pathophysiology of ischemic stroke; numerous studies have indicated that plasma levels of IL-6, C-reactive protein and other pro-inflammatory cytokines correlate with brain infarct volume and stroke severity and are early markers for long-term outcomes in AIS patients [18–20]. Our data also showed a strong association between the serum exosomal levels of miR-9, miR-124 and IL-6 (Fig 5), further suggesting that serum exosomal miRNAs may provide diagnostic and prognostic information for patients with acute ischemic stroke.

Circulating miRNAs exist in two different package forms, either being encapsulated in microvesicles (exosomes and shedding vesicles) or being associated with proteins of Argonaute family or HDL particles. In 2007, Veladi and colleagues demonstrated that miRNA and mRNA can be exchanged between cells by exosomes and subsequently exert their particular biological functions [8], but there is still no such evidence for the vesicle-free miRNA. Therefore, serum exosomes are not only a valuable source of potential biomarkers [35], but also an important factor in the horizontal transfer of biological information. A recent study by Ridder's group has validated the exosomal miRNAs mediating remote communication. The authors have found that MVs derived from hematopoietic cells can be taken up by cells in many different organs, such as the liver, lung and small intestine. Intriguingly, these MVs can transfer the genetic information from the hematopoietic system to brain cells under conditions of inflammation [36]. Their study has unraveled a novel communication mechanism between the hematopoietic system and various organs by exosomes.

Given the important roles of exosomes in mediating intercellular communication, and the functions of miR-9 and miRNA-124 in anti-inflammatory action by inhibiting the production of pro-inflammatory cytokines [37, 38], exosomes derived from brain tissues may be absorbed by circulating immune cells, such as monocytes, and modulate their phenotype towards immunological suppression. Currently, post stroke immunosuppression, manifested by systemic infections such as pneumonia and urinary tract infections, is believed to be mediated by catecholamines and steroids released by sympathetic activation after a stroke [39]. More research efforts are needed to reveal whether leakage of these brain tissue derived exosomes is involved in post stroke immunosuppression.

Altogether, in this proof of concept study, our data revealed significantly elevated levels of serum exosomal miR-9 and miR-124 in AIS patients, and they are promising biomarkers for diagnosing AIS and evaluating the degree of damage caused by the ischemic injury. However, multicenter studies are needed to confirm the specificity and sensitivity of these circulating miRNAs. In addition, it will be worthwhile to explore the potential biological effects of these sudden intruding brain-derived exosomes, which transfer information from the central nervous system to the peripheral system, and their involvement in post-stroke complications.

## Supporting Information

S1 Fig Correlations between the NIHSS scores and infarct volumes. (TIFF)

S1 Table Detailed information of 65 AIS patients and 66 healthy controls. (XLS)
