# Supplementary figures and images for: Increased Brain-Specific MiR-9 and MiR-124 in the Serum Exosomes of Acute Ischemic Stroke Patients
Source: PLoS One. 2016 Sep 23;11(9):e0163645. doi: 10.1371/journal.pone.0163645 (PMC5035015; doi:10.1371/journal.pone.0163645)

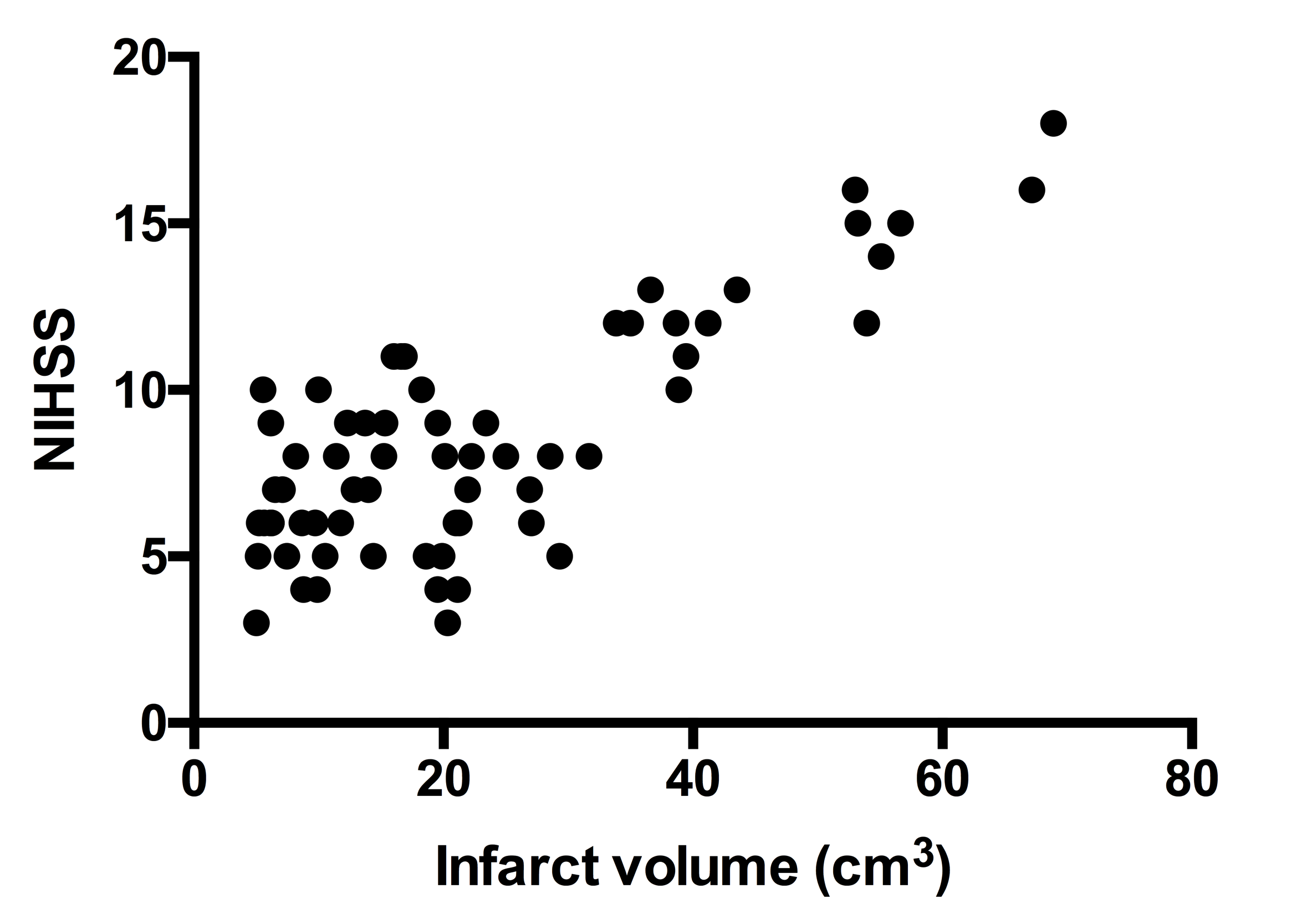

Supplement: S1 Fig — (TIFF) [file pone.0163645.s001.tiff]
